# Transcriptomic and Proteomic Spatial Profiling of Pediatric and Adult Diffuse Midline Glioma H3 K27-Altered, Reveals Region Specific Differences and Limited Overlap between mRNA and Protein

**DOI:** 10.21203/rs.3.rs-4139314/v1

**Published:** 2024-04-05

**Authors:** Sudarshawn Damodharan, Jack M. Shireman, Elliot Xie, Emily Distler, Christina Kendziorski, Mahua Dey

**Affiliations:** Ann & Robert Lurie Children’s Hospital of Chicago; University of Wisconsin School of Medicine & Public Health; University of Wisconsin School of Medicine & Public Health; University of Wisconsin School of Medicine & Public Health; University of Wisconsin School of Medicine & Public Health; University of Wisconsin School of Medicine & Public Health

## Abstract

Diffuse midline glioma, *H3 K27*-altered (DMG-Alt) are highly aggressive malignancies of the central nervous system (CNS) that primarily affect the pediatric population. Large scale spatial transcriptomic studies have implicated that tumor microenvironmental landscape plays an important role in determining the phenotypic differences in tumor presentation and clinical course, however, data connecting overall transcriptomic changes to the protein level is lacking.

The NanoString GeoMx^™^ Digital Spatial Profiler platform was used to determine the spatial transcriptomic and proteomic landscape in a cohort of both pediatric and adult *H3 K27*-altered DMG biopsy samples. Three fluorescently labeled antibodies targeting immune cells (CD45), epithelial cells (PanCK), tumor cells *(H3 K27M)* and a nucleic acid stain (SYTO-13) were used to establish regions of interest (ROI) for genomic and proteomic analysis.

We found genetic alterations within the tumor which can be delineated across patient age and spatial location. We show that the H3 K27M mutation itself has a profound impact on tumor cells transcriptomics and interestingly we found limited fidelity between overall transcriptome and proteome. Our data also validate the previously described OPC like precursor signature at the proteomic level and reveal a special shift in the signature based on the local TME composition.

## Introduction

Diffuse midline glioma *H3 K27*-altered is a fatal central nervous system (CNS) malignancy that predominantly affects children of age 5–7 years. it is classified as a World Health Organization (WHO) grade 4 neoplasm with no curative treatments. The current standard approach to treatment is radiation therapy which is only palliative. The incidence rate for these tumors is difficult to accurately quantify due to the recent change in molecular classification as well as the rarity of cases. The most current incidence data collected by the World Health Organization (WHO) reports an incidence rate of 0.54 cases per million person years in adults, and a rate of 2.23 cases per million person years in people ≤ 20 years of age^1,2^. The prognosis of this neoplasm is extremely poor, especially in children with a 2-year survival rate of < 10%^1,2^. Due to the rarity of the tumors in adults and the many subclassifications of DMG’s, true prognosis in adults is still debated; however, it’s generally agreed that adults have a better, while still overall poor, clinical prognosis^3–5^. These neoplasms occur primarily in midline structures within the CNS, with the brainstem and thalamus being the most prevalent and equally fatal anatomical locations^6–9^. Due to the critical anatomical locations of DMGs, surgical resection or needle biopsies were traditionally not performed, limiting the amount of pathological and molecular data obtained to date. However, with the development of sophisticated surgical technology over the last decade, biopsy has become routine resulting in a better understanding of DMGs at a genomic and molecular level.

These advanced molecular studies have led to further classification of DMGs into DMG H3K27-altered (DMG-Alt) based on specific histone mutations^10,11^ the most significant being highly recurrent histone mutations *(H3F3A* or *HIST1H3B/C)* which are detected mostly in pediatric cases^7,12,13^. Liu et al provided the first look into the genomic landscape of DMG-Alt at a spatial level across varying age and anatomical location^14^. In doing so, oligodendroglial lineage of precursor tumor cells were identified to be the majority in all clinical and anatomical groups and mesenchymal precursor signatures were increased in older patients with DMG-Alt^14^. Our understanding of the molecular and genetic landscape of these malignancies continues to grow dramatically, but primarily at the mRNA and transcriptomic level^10–12,14^ downstream proteomic concordance of the transcriptomic landscape has yet to be evaluated.

Although the mRNA landscape provides a good overview of the overall genomic alteration of a malignancy, it is the overall functional proteins that are responsible for the pathological behavior of the malignancy. Translation of mRNA into protein is a complex process with varying levels of translational fidelity between mRNA and protein^15–17^. Correlations between the differential expression of specific mRNAs and their corresponding proteins have been assessed in varying human disease processes with varying levels of concordance^18–23^. The fidelity between transcriptomic and proteomic landscape has never been explored in DMG-Alt.

To address these gaps in the literature we initiated this study in which we utilized spatial multi-omics profiling to comprehensively evaluate the molecular landscape of a cohort of pediatric and adult DMG-Alt tumor samples.

## Methods

### Clinical Sample Processing:

**Figure 1:**
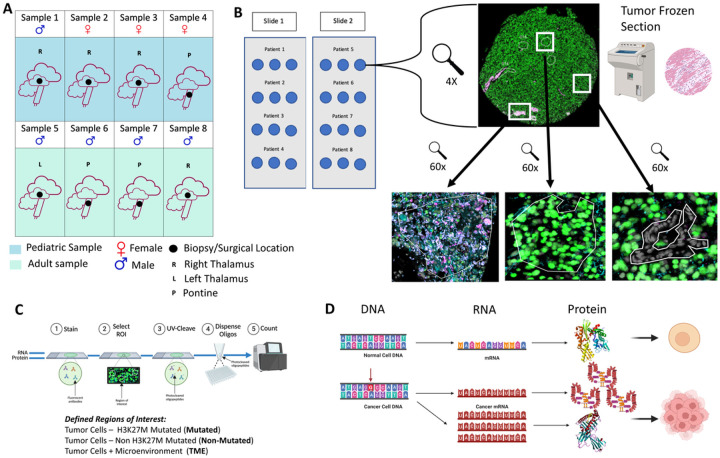
Patient cohort and experimental workflow: **A**) Graphic depicting the tumor location, patient age, and location of the tissue biopsy analyzed for the study. **B**) Graphic depicting the slide layout of the Nanostring GeoMx slide used for sequencing. **C**) Cartoon illustrating the experimental workflow of the project and how the ROIs were defined. **D**) Cartoon depicting the lack of translation fidelity in cancer compared to normal cells.

Tissue samples (n = 8) and clinical data was collected from previously archived formalin-fixed-paraffin-embedded tissue (FFPE) samples of both pediatric (n = 4) and adult (n = 4) patients with confirmed pathological diagnosis of DMG, *H3 K27*-altered. IRB approval was obtained to utilize the archived pediatric and adult pathological samples for analysis at the University of Wisconsin-Madison (2022–0164-CP001). All patients analyzed within the study provided informed consent and all analyses were carried out according to UWSMPH/UW-Madison IRB direction and supervision. All samples were obtained from biopsies or resections done pre-treatment at the time of initial diagnosis. From each sample, three cores were obtained to have each patient sample represented as a triplicate to evaluate the broader tumor landscape and proteogenomic profiles. (Fig. 1A & B, Table 1, Sup Fig. 1). All analysiss conducted on human samples was performed in accordance with relevant guidelines and regulations.

### Spatial Genomic Profiling:

Transcriptomic and proteomic profiling was done on the above samples. The anatomical locations included the thalamus (n = 5) and pons (n = 3). To evaluate the intra-tumoral landscape and heterogeneity, the samples were stained with antibodies against the epithelial cell marker, PanCK; the leukocyte marker, CD45; mutant-tumor specific marker against H3 K27M; and the nuclear stain SYTO13. We performed spatial transcriptomic and proteomic characterization of all samples via *in situ* sequencing utilizing the Nanostring digital spatial profiling (DSP) system and Illumina sequencing platforms (Fig. 1B & C). Across all samples, a total of 49 ROIs were marked for this experiment; divided between both populations of samples (25 for pediatric and 24 for adult). Each ROI accounted for approximately 500uM of total space with the differing cells encompassed within this. The ROI categories utilized for this experiment were H3 K27M Ab predominant, H3 K27 ab + CD45 and areas predominantly with unlabeled tumor cells (non-H3 K27M positive tumor cells). Each ROI was assessed for the composition of mutant neoplastic cells, immune cells and interactions with one another.

### ROI Collection and Filtering:

A total of 94 ROIs were initially collected for analysis (Fig. 1C). The subsequent filtering process, which incorporated criteria such as gene detection rates, total nuclei count, and the intrinsic relevance of each ROI type, resulted in 85 ROIs being selected for further analysis. To ensure a robust and meaningful deconvolution analysis, necessitating sufficient diversity among the ROIs, a more stringent filtering criterion based on gene detection rates was applied, ultimately retaining 49 ROIs for deconvolution analysis.

### Annotation of ROIs:

Each ROI underwent several runs of manual annotation. This involved an in-depth examination of the corresponding-colored images and the screening of highly expressed protein markers characteristic of each region.

### Correlation Analysis between Protein and RNA:

For the correlation analysis, proteins and RNA were matched according to their respective GeneIDs. A Pearson correlation coefficient was calculated for each pair to assess the degree of linear correlation between the protein and RNA expression levels in the ROIs (Fig. 1D). Hypothesis testing to identify differences between average correlations in adult vs. pediatrics (and in mutated vs. non-mutated vs. TME) was conducted by transforming correlations using Fisher’s Z-transformation and evaluating p-values via Students t-test (ANOVA).

### Deconvolution Analysis:

Deconvolution of the spatial data was carried out using the SpatialDecon algorithm^24^. This method utilizes log-normal regression and integrates background modeling, thereby enhancing performance beyond that of traditional least-squares methods. SpatialDecon is adept at quantifying cell populations within ROIs and provides precise estimates of cell abundance. The reference matrix, crucial for this analysis, was sourced from a study focused on DMG single-cell RNA sequencing data^14^. The processing of this data was in strict accordance with the protocols and methodologies detailed in Liu et al., 2022^14^. For comparisons among the SpatialDecon identified regions a Wilcoxon ranked tested adjusted for multiple comparisons was utilized with adjusted p values reported. 0.

### Gene Regulatory Network Reconstruction and Cell-State Identification:

To comprehensively characterize the transcriptional state of each ROI, we utilized SCENIC^25^, a computational approach for concurrent gene regulatory network reconstruction and cell-state identification. The SCENIC methodology involves three steps: initially, GRNboost2 identifies gene sets co-expressed with transcription factors. Subsequently, these gene sets are refined through cis-regulatory motif analysis using RcisTarget, leading to the formation of regulons by retaining modules with significant motif enrichment. The activity of each regulon in individual ROIs is quantified using the AUCell algorithm, yielding an AUC score that reflects the subnetwork activity. This score is then utilized to assess the characteristics of each ROI.

#### Over-representation Analysis:

Over-representation analysis (ORA) was done using webgestalt^26–28^ with default parameters on functional database “gene ontology” or biological pathways “KEGG”. Enrichment statics are calculated using a hypergeometric test to evaluate the significance of enrichment and calculate a p-value which is then adjusted for multiple comparisons as well as a false discovery rate (FDR)^26–28^. For this study FDR is reported for all ORA comparisons made with significance being considered if FDR < 0.10.

## Results

### Patient Cohort and Clinical Characteristics:

Patient characteristics along with treatment details are listed in Table 1 and Fig. 1A along with experimental details and schematics (Fig. 1B, C, & D). All patients in the study had mutations within the *H3F3A* gene which encodes histone H3.3 and typically correlates with a more severe phenotype in this tumor^5^. For the pediatric patients, the median age at diagnosis was 8.5 (range: 3–14 years); median overall survival (OS) was 9.2 months (range: 2–14 months). For the adult patients, the median age of diagnosis was 40.5 (range: 25–48); median overall survival (OS) was 14.2 months (range: 1–26 months)

### SCENIC profiling reveals spatially differential regulon enrichment:

**Figure 2:**
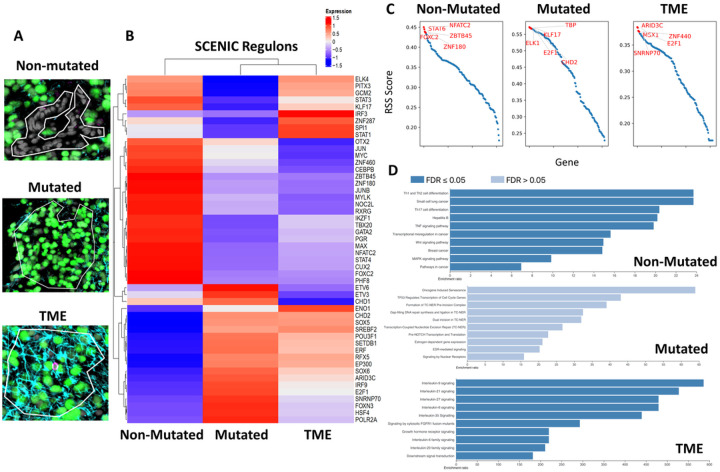
SCENIC profiling reveals spatially differential regulon enrichment: **A**) IHC representations of the composition of the selected ROIs analyzed by SCNEIC. **B**) SCENIC regulon enrichment across our selected ROIs visualized using a heatmap which includes the set of significantly enriched terms for each ROI. **C**) Regulon Specificity Score (RSS) across the analyzed ROIs with the top genes highlighted in red. **D**) Over representation analysis of the genes contained within the SCENIC enrichment for each ROI analyzed. Statistical comparisons made within SCENIC using RSS score with FDR reported in the legend.

SCENIC was used to reconstruct gene regulatory networks and estimate cellular states for each ROI. The combined adult and pediatric samples were classified spatially by grouped ROIs into 3 categories: H3K27M mutated tumor cells (Mutated), Non H3K27M mutated tumor cells (Non-Mutated), and tumor cells + surrounding microenvironment (TME). These ROIs were confirmed using IHC staining on our tissue samples, allowing for anchoring and targeting of the resulting genomic and proteomic analysis done by the GEOMX system. Mutated ROIs contained only cells expressing H3K27M marks (green), while TME ROIs contained tumor cells (green), immune cells (pink), and endothelial cells (blue) (Fig. 2A). ROIs containing cells with no staining (Non-Mutated) were hypothesized to contain mostly tumor cells that do not carry the H2K27m mutation which is seen in the tumor bulk according to recent reports^8,29^. To investigate this hypothesis, the top enriched genes/regulons identified using SCENIC were subject to over representation analysis (ORA) and compared to an equal number of enriched genes/regulons derived from a dataset of normal thalamic and pontine tissue (http://www.brain-map.org/). This comparison demonstrated highly enriched signaling for cancer associated genes and tumor suppressors with enriched terms such as Transcriptional Regulation in Cancer and Pathways in Cancer (FDR < 0.05). Conversely, ORA analysis on the normal thalamic and pontine tissue showed typical enrichment for cell developmental processes and neuronal signatures with enrichment terms for regulation of synaptic signaling and mononuclear cell proliferation (FDR < 0.01) (Sup Fig. 2A & B). Across our spatial classifications, SCENIC identified significantly different regulons driven by ARID3C and MSX1 in the TME, TBP and ELK1 in Mutated, and NFATC2 and Stat6 in Non-mutated ROIs (Fig. 2B & C). These regulons? were enriched for cytokine and interleukin signaling in the TME ROIs (FDR < 0.05), cancer induced senescence and TP53 gene regulation in the Mutated ROIs (FDR < 0.10), and TNF and WNT pathway signaling in Non-Mutated ROIs (FDR < 0.05) (Fig. 2D). These results highlight that spatially distinct regions within the tumor rely on differential transcriptional programs especially with regards to invading immune cells and surrounding vasculature. Furthermore, the H3K27 mutation itself seems to impact the transcriptional landscape significantly with non-mutated tumor cells displaying more developmental like signaling pathways as opposed to mutated tumor cells displaying more canonical cancer signaling networks.

### OPC like cell signature varies across age and spatial location:

**Figure 3:**
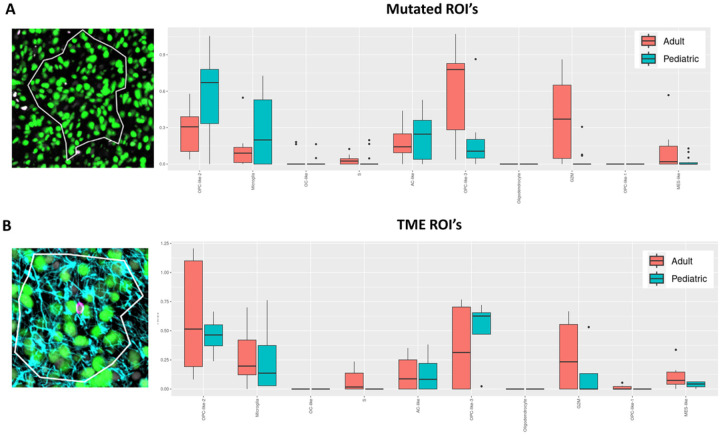
OPC like cell signature varies across age and spatial location: **A)** Visual representation of the Mutated ROI used for deconvolution analysis as well as a histogram of the results of analysis comparing the adult (red) and pediatric (blue) populations within the Mutated ROI. **B**) Visual representation of the TME ROI used for deconvolution analysis as well as a histogram of the results of analysis comparing the adult and pediatric populations within the TME ROI.

Molecular and genomic classification of DMG-Alt remains in its infancy due to the extremely limited number of available patient samples compared to other glioma types. In a recently published seminal study^14^ the authors describe a ubiquitous oligodendrocyte precursor cell (OPC) like cellular state across both pediatric and adult DMG-Alt. Other cell states identified in the tumor include Mesenchymal (Mes) like and Astrocytic (AC) like, as well as cycling cells. Interestingly, neuronal signatures are not seen within DMGs from either population. We validated this in our dataset as well where we only saw enrichment for neuronal signatures during ORA analysis of normal midbrain controls (Sup Fig. 2A & B). Utilizing their published datasets, we compared our samples across age and spatial location and were able to corroborate the baseline OPC like signatures seen in adults and pediatrics. In our data examining Mutated ROIs we found pediatric tumors enriching for OPC like (specifically OPC like 2) and microglial signatures while adult tumors enrich for Mes like and AC signatures (Fig. 3A). Interestingly, when we examined TME ROIs we saw a shift in the observed signatures with adult tumors now showing increased OPC like signature enrichment as well as a stronger microglial signature (peds vs adults: padj < 0.05 vs padj = 1.0) (Fig. 3B).

These genomic signatures were originally derived from RNA data. However, our dataset includes a subset of protein targets allowing for partial validation of the expression pattern for one of the component genes of the OPC like signature, EGFR (Sup Fig. 3) at the protein level. These results indicate that even though our study has a relatively limited sample size, it still captures and validates a previously published genomic classification across patient age using both RNA and protein expression data. Our results also highlight a spatially driven shift in these signatures when more complex cell compositions are examined. This could indicate a TME driven shift in the tumor cells’ biology or simply be reflective of the more complex TME present along outer edges of tumor compared to the tumor cell dense cores.

### Correlation between RNA and Protein varies both spatially and across patient age:

**Figure 4:**
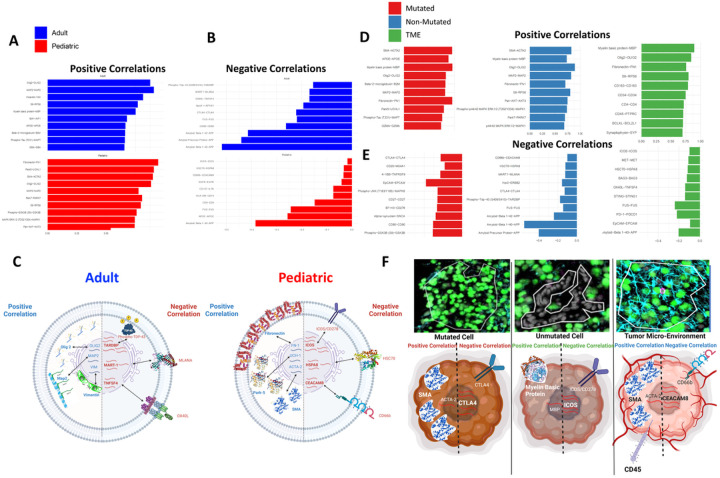
Correlation between RNA and Protein varies both spatially and across patient age: **A**) Histogram representations of the top 10 positively correlated RNA/protein pairs across adults (blue) or pediatric samples (red). **B**) Histogram representations of the top 10 negatively correlated RNA/protein pairs across adults (blue) or pediatric samples (red). **C**) Cartoon representation of translational fidelity using the top 3 positive or negatively correlated RNA/protein pairs in adult or pediatric samples. **D**) Histogram representations of the top 10 positively correlated RNA/protein pairs across all 3 ROIs contained in our sample population. **E**) Histogram representations of the top 10 negatively correlated RNA/protein pairs across all 3 ROIs contained in our sample population. **F**) Cartoon representation of translational fidelity using top positive or negatively correlated RNA/protein pairs in cells from each ROI contained within our sample population.

A large portion of the current genomics work assumes that increased mRNA abundance results in increased protein abundance. While this is often the case, it is well known that the amount of translated protein can vary widely from the amount of detected mRNA^30–32^. Leveraging the unique Nanostring GeoMx technology we detected both RNA and protein probes across our entire range of samples. This allowed for comparison of matched RNA to protein pairs to assess transcription to translation fidelity and characterize the extent to which it varies across our samples. We first visualized the correlation between mRNA and protein abundance (ranging from – 1 being 100% negative correlation to 1 being 100% positive correlation) across the adult and pediatric tumor samples. We found similar rates of correlation on average in adults (0.19 +/− 0.27 SD) and pediatrics (0.33 +/− 0.25 SD) (Sup Fig. 4A) but differences were observed among the top 10 gene pairs that are most (and least) correlated (Fig. 4A, B, & C). Spatially, similar correlation results were observed with no statistical difference in correlation rates (mutated vs non-mutated vs TME: 0.28 +/− 0.31SD, 0.24 +/− 0.26SD, 0.29 +/− 0.27 SD; p > 0.05) (Sup Fig. 4B), however, differences among the specific gene pairs that were correlated vs. anticorrelated were seen (Fig. 4D, E, & F). Across adult and pediatric samples positive correlation values between RNA and protein were similar for some genes (OLIG2, Vimentin, MAP2) but differential correlation was also seen in adults and pediatric samples. Spatially, similar proteins were observed positively correlated across regions that were also observed across ages (OLIG2, MBP MAP2). In terms of negative correlation CTLA4 was a top negative correlation across both age and spatial ROIs, an interesting finding due to the relevance of CTLA4 treatments across many other cancer types^33–35^. Overall, these results show that not only are different transcriptional programs being activated in tumors across age and regions, but these transcriptional programs do not always faithfully lead to the corresponding proteins being translated. If translational fidelity is not considered, predictions biased towards RNA content, but not true protein translation, may result.

### Common genomic therapeutic targets show differing RNA to protein correlation:

**Figure 5:**
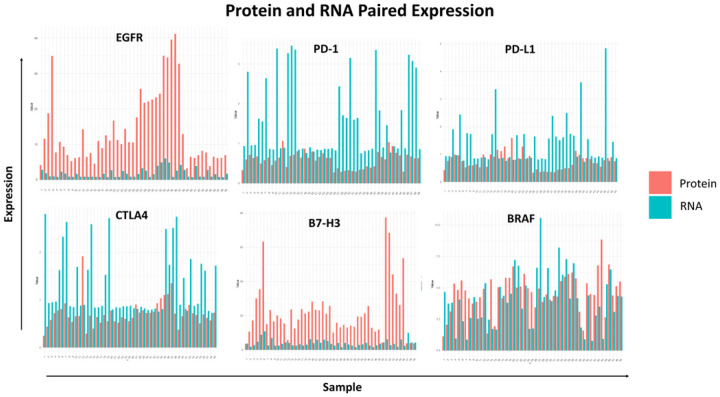
Common genomic therapeutic targets show differing RNA to protein correlation: Histogram representation of expression of RNA (blue) and protein (red) levels across all samples for common genomic targets of DMG-Alt.

Much of the upcoming targeted and immunotherapeutic interventions that are being studied for DMG-Alt have come about based upon previously established transcriptomic information. However, downstream proteomic data of clinically relevant therapeutic targets have not been readily studied. To examine this in our dataset we found RNA-Protein pairs that represented promising or already tried therapeutic targets in this group of malignancy and quantified their RNA and protein levels across all samples. Expression of receptor tyrosine kinase (RTK) has been shown to be prevalent on these malignancies with on-going and previously completed studies examining EGFR as a relevant target both as a small molecule inhibitor and in combination with immunotherapeutic agents^36–38^. Interestingly, low concordance was observed for EGFR between RNA and protein, with protein levels higher than would have been predicted by mRNA abundance (Fig. 5). Checkpoint blockade has also been tested both as the monotherapy and combinatorial immunotherapy for this group of malignancy^39–41^, thus we examined PD1, PDL-1 and CTLA-4 expression correlation in our dataset. We observed low concordance between RNA and protein across all the above markers with CTLA-4 especially enriched for negative correlation when examined in our TME ROIs (see Fig. 4E). For these molecules, the amount of RNA detected was more than the actual amount of protein detected which could lead to skewed optimistic prediction of outcome for therapeutic targeting in clinical settings. B7-H3 and BRAF have been well characterized across tumors and within our dataset we found close concordance for RNA and protein levels for BRAF but not B7-H3 with the latter producing more protein than detected RNA. To further explore more clinically relevant targets with our data we filtered GSEA enrichment results using Enrichr^42^ for genes involved in pathways that resulted in molecular secretions. The results demonstrated the top enriched pathways belonging to IL6 and ATF2 signaling events with glucocorticoid receptor signaling and calcium signaling also being present, likely related to the very neuronally active locations of these tumors (Sup Fig. 5). Together, these results highlight the crucial point that extensive targeting solely based on transcriptomics may result in false positives as well as false negatives and integrating proteomic data may strengthen early-stage clinical drug screening.

## Discussion

DMG-Alt continues to be a devastating disease with limited therapeutic options, however, in the last decade neurosurgical and neuro-oncological techniques have advanced considerably allowing for biopsy and sample collection from these tumors. This in turn has led to several novel clinical trials currently underway based on the genomic targets discovered from comprehensive transcriptomic studies. Our study aims to push the field forward by taking the next step in genomic screening by examining spatial aspects of the tumor architecture as well as proteomic profiles. Spatially, we found that tumor ROIs that contain portions of TME display distinct genetic signatures compared to regions with dense tumor cell populations. Using proteomics, we demonstrate that transcription to translation fidelity is not always maintained in both adult and pediatric DMG-Alt. Finally, we interrogated common genetic targets proposed both for these tumors as well as broadly used in other gliomas and reveal the shortfalls of predicting therapeutic targeting efficacy based on RNA data alone. Continued research in this field will be crucial in advancing both the molecular and clinical understanding of these rare and lethal tumors.

A recent publication by Liu and colleagues demonstrated an increased tendency for pediatric DMG-Alt to be dominated by an OPC like cell type while adult DMG-Alt enriched for a mesenchymal cell phenotype^14^. In our cohort we saw similar patterns across adult and pediatric tumors, however when examining the spatial landscape, we observed a change in the signature in ROIs composed of both tumor and TME cells. Also of note, in both our cohorts of data there is little to no neuronal signature expressed within any sample set. This is interesting because these tumors arise in very neuronally dense and eloquent tissue but seem to not be as intermixed with their surroundings like glioblastomas (GBM), the most common primary CNS malignancy in adults, which are known to integrate into neuronal synapses and proliferate more in response to neuronal firing^43–46^. This may indicate that DMG-Alts interacts more with other brain resident cells such as microglia, astrocytes, or oligodendrocytes as opposed to neurons. It’s also worth noting that there was little to no outside immune cell infiltration into the tumor bulk itself which is consistent with reports of DMG-Alt being an immune desert^47,48^.

Our study is the first to include spatial proteomic profiling and we demonstrate that while translational fidelity was measured at similar levels across adults and pediatric samples, as well as across the ROIs, there were subtle differences in the types of genes that were either faithfully or not faithfully translated from mRNA. These results have significant implications for cancer therapeutics in general which to date has relied mostly on screening mRNA due to difficulties in obtaining proteomic data. Our examination of common genomic targets discovered using mRNA alone shows that although this method can result in true proteomic hits there is also room for error including both false positive and false negative hits being prioritized (such as EGFR false negative vs. PD-1 false positive). Although there are some studies suggesting mRNA, specifically differentially expressed mRNA, may be a faithful readout of true protein synthesis^49^, this has yet to be widely examined in the context of brain malignancies, especially in the pediatric population.

One of the limitations of our study is that the number of protein probes available is far less than the number of RNA probes, and so a genome-wide comparison of RNA to protein was not possible. We also did not have a large enough number of samples to include statistically significant sex comparisons. In addition, due to the technical limitations of the Nanostring platform regarding the number of antibodies that can be used for defining ROIs, we had a subset of cells that were unstained. Since all our samples were targeted needle biopsies aimed at obtaining the most pathological tumor tissue, we hypothesized that these were most likely tumor cells that lacked the H3K27m mutation. In validating this using ORA analysis, we discovered a unique genetic program within these cells compared to H3K27m tumor cells involving some immune signatures as well as cancer and developmental gene enrichment. Although we only visualize a limited number of immune cells in our samples, there were samples with strong enrichment for immune related signaling, especially when TME ROIs were considered. This may be a result of tumor cells hijacking immune signaling methods (seen in other gliomas^50–52^) or could result from immune cells sitting mostly on the peripheral edges of the tumor (which would not be captured during biopsy) inducing inflammation. An in depth understanding of the immune response and its resulting inflammation in this context may prove critical as we try to advance therapies, particularly CAR-T cell based therapies which are currently undergoing clinical trials^53–55^.

Although DMG-Alt remains a 100% lethal tumor with incredibly limited survival, novel research is starting to illuminate its genetic background and expose possible therapeutic vulnerabilities. Further studies across multiple institutions to increase sample size will be the key in validating and advancing effective therapies and ultimately making a difference in patient survival.

## Figures and Tables

**Table 1:** Clinical characteristics of pediatric and adult patients

Patient	Age at Diagnosis (years)	Sex	Race	Location	Treatment	OS (months)
1	6	Male	Caucasian	Right Thalamus	XRT + TMZ, ONC201	14
2	11	Female	Hispanic	Right Thalamus	XRT	9
3	14	Female	Black	Right Thalamus	XRT	12
4	3	Female	Black	Pontine	XRT	2
5	43	Male	Caucasian	Pontine	XRT	6
6	46	Male	Hispanic	Left Thalamus	None	1
7	48	Male	Caucasian	Pontine	XRT + TMZ, ONC201	26
8	25	Male	Black	Right Thalamus	XRT + bevacizumab	24

XRT – radiotherapy, OS – overall survival, TMZ – temozolomide

## Data Availability

All sequencing raw and processed data will be deposited into GEO and released publicly upon publication of the manuscript. The datasets used and/or analyzed during the current study are available from the corresponding author on reasonable request.
